# *N*,*N*′-Bis(4-methyl­phenyl­sulfon­yl)­suberamide

**DOI:** 10.1107/S1600536811029783

**Published:** 2011-07-30

**Authors:** Vinola Z. Rodrigues, Sabine Foro, B. Thimme Gowda

**Affiliations:** a Department of Chemistry, Mangalore University, Mangalagangotri 574 199, Mangalore, India; b Institute of Materials Science, Darmstadt University of Technology, Petersenstrasse 23, D-64287 Darmstadt, Germany

## Abstract

In the crystal structure of the title compound, C_22_H_28_N_2_O_6_S_2_, the asymmetric unit contains one half mol­ecule with a center of symmetry at the mid-point of the central C—C bond. The conformations of all the N—H, C= O and C—H bonds in the central amide and aliphatic segments are *anti* to their adjacent bonds. The mol­ecule is bent at the S atom with an C—SO_2_—NH—C(O) torsion angle of −76.4 (3)°. The dihedral angle between the benzene ring and the SO_2_—NH—C(O) segment in the two halves of the mol­ecule is 67.2 (1)°. In the crystal, N—H⋯O(C) inter­molecular hydrogen bonds link the mol­ecules into chains along the *b* axis.

## Related literature

For studies on the effects of substituents on the structures and other aspects of *N*-(ar­yl)-amides, see: Arjunan *et al.* (2004 ▶); Gowda *et al.* (1999 ▶, 2006 ▶); for *N*-(ar­yl)-methane­sulfonamides, see: Gowda *et al.* (2007 ▶); and for *N*-(aryl­sulfon­yl)-amides, see: Rodrigues *et al.* (2011 ▶)
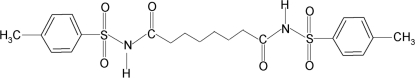


## Experimental

### 

#### Crystal data

C_22_H_28_N_2_O_6_S_2_
*M* *_r_* = 480.58
Monoclinic, 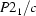

*a* = 8.025 (1) Å
*b* = 15.835 (2) Å
*c* = 10.106 (1) Å
β = 112.31 (1)°
*V* = 1188.1 (2) Å^3^
*Z* = 2
Mo *K*α radiation
μ = 0.26 mm^−1^
*T* = 293 K
0.22 × 0.20 × 0.06 mm

#### Data collection

Oxford Diffraction Xcalibur diffractometer with a Sapphire CCD detector
Absorption correction: multi-scan (*CrysAlis RED*; Oxford Diffraction, 2009 ▶) *T*_min_ = 0.944, *T*_max_ = 0.984
4285 measured reflections
2174 independent reflections
1326 reflections with *I* > 2σ(*I*)
*R*_int_ = 0.027

#### Refinement

*R*[*F* ^2^ > 2σ(*F* ^2^)] = 0.058
*wR*(*F* ^2^) = 0.139
*S* = 1.04
2174 reflections
149 parameters
1 restraint
H atoms treated by a mixture of independent and constrained refinement
Δρ_max_ = 0.24 e Å^−3^
Δρ_min_ = −0.18 e Å^−3^

### 

Data collection: *CrysAlis CCD* (Oxford Diffraction, 2009 ▶); cell refinement: *CrysAlis RED* (Oxford Diffraction, 2009 ▶); data reduction: *CrysAlis RED*; program(s) used to solve structure: *SHELXS97* (Sheldrick, 2008 ▶); program(s) used to refine structure: *SHELXL97* (Sheldrick, 2008 ▶); molecular graphics: *PLATON* (Spek, 2009 ▶); software used to prepare material for publication: *SHELXL97*.

## Supplementary Material

Crystal structure: contains datablock(s) I, global. DOI: 10.1107/S1600536811029783/nc2239sup1.cif

Structure factors: contains datablock(s) I. DOI: 10.1107/S1600536811029783/nc2239Isup2.hkl

Supplementary material file. DOI: 10.1107/S1600536811029783/nc2239Isup3.cml

Additional supplementary materials:  crystallographic information; 3D view; checkCIF report

## Figures and Tables

**Table 1:** Hydrogen-bond geometry (Å, °)

*D*—H⋯*A*	*D*—H	H⋯*A*	*D*⋯*A*	*D*—H⋯*A*
N1—H1*N*⋯O3^i^	0.85 (2)	2.12 (2)	2.968 (3)	177 (3)

Symmetry code: (i) 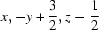.

**Fig. 1.**
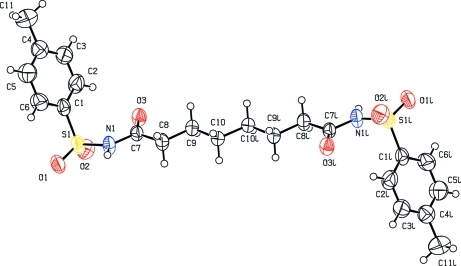
Molecular structure of the title compound, showing the atom labelling scheme. Displacement ellipsoids are drawn at the 50% probability level and H atoms are represented as small spheres of arbitrary radii (Symmetry code: (i) -x + 2, -y + 2, -z + 1).

**Fig. 2.**
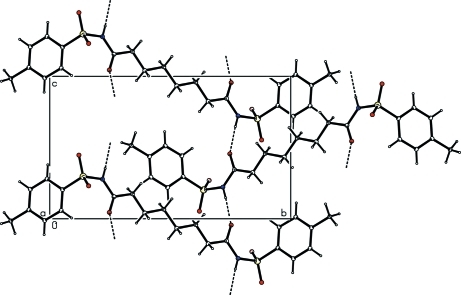
Molecular packing of the title compound with hydrogen bonding shown as dashed lines.

## supplementary crystallographic information

### Comment

The amide and sulfonamide moieties are important constituents of many biologically significant compounds. As part of our studies on the effects of ring and side chain substitutions on the structures and other aspects of *N*-(aryl)-amides (Arjunan *et al.*, 2004; Gowda *et al.*, 1999, 2006), *N*-(aryl)-methanesulfonamides (Gowda *et al.*, 2007) and *N*-(arylsulfonyl)-amides (Rodrigues *et al.*, 2011), the crystal structure of *N*,*N*-bis(4-methylphenylsulfonyl)-suberamide (I) has been determined (Fig. 1).

In the two C—SO_2_—NH—CO—CH_2_—CH_2_—CH_2_— central segments of the structure, all the N—H, C=O and C—H bonds in the amide and aliphatic segments are *anti* to the adjacent bonds, similar to that observed in *N*,*N*-bis(4-chlorophenylsulfonyl)-suberamide (II) (Rodrigues *et al.*, 2011). The orientations of sulfonamide groups with respect to the attached phenyl rings are given by the C2—C1—S1—N1 and C6—C1—S1—N1 torsion angles of 78.2 (4)° and -104.3 (3)°, respectively, compared to the corresponding angles of 67.2 (3)° and -113.9 (4)° in (II).

The molecule is bent at the S atom with the C1—S1—N1—C7 torsion angle of -76.4 (3)°, compared to the value of -80.6 (4)° in (II). In (I), the aliphatic chain is almost linear with C7—C8—C9—C10 torsion angle of -176.9 (3)°, compared to the value of -179.4 (4)° in (II).

The dihedral angle between the benzene ring and the SO_2_—NH—C(O) segment in the two halves of the molecule is 67.2 (1)°, compared to the value of 79.5 (2)° in (II).

The structure shows simultaneous of N—H···O(C) and N—H···O(S) intermolecular hydrogen bonds (Table 1), which link the molecules into infinite chains along the *b*-axis.

### Experimental

*N*,*N*-Bis(4-methylphenylsulfonyl)-suberamide was prepared by refluxing a mixture of suberic acid (0.01 mol) with 4-methylbenzenesulfonamide (0.02 mol) and POCl_3_ (0.02 mol) for 1 h on a water bath. The reaction mixture was allowed to cool and added ether to it. The solid product obtained was filtered, washed thoroughly with ether and hot ethanol. The compound was recrystallized to the constant melting point and was characterized by its infrared and NMR spectra.

Plate like colorless single crystals used in the X-ray diffraction studies were grown by a slow evaporation of a solution of the compound in ethanol at room temperature.

### Refinement

The H atom of the NH group was located in a difference map and later restrained to the distance N—H = 0.86 (2) Å. The other H atoms were positioned with idealized geometry using a riding model with the aromatic C—H = 0.93 Å, the methyl C—H = 0.96Å and the methylene C—H = 0.97 Å. All H atoms were refined with isotropic displacement parameters. The *U*_iso_(H) values were set at 1.2*U*_eq_(C-aromatic, N) and 1.5*U*_eq_(*C*-methyl).

### Crystal data

**Table:**

C_22_H_28_N_2_O_6_S_2_	*F*(000) = 508
*M**_r_* = 480.58	*D*_x_ = 1.343 Mg m^−^^3^
Monoclinic, *P*2_1_/*c*	Mo *K*α radiation, λ = 0.71073 Å
Hall symbol: -P 2ybc	Cell parameters from 1291 reflections
*a* = 8.025 (1) Å	θ = 2.6–27.9°
*b* = 15.835 (2) Å	µ = 0.26 mm^−^^1^
*c* = 10.106 (1) Å	*T* = 293 K
β = 112.31 (1)°	Plate, colourless
*V* = 1188.1 (2) Å^3^	0.22 × 0.20 × 0.06 mm
*Z* = 2	

### Data collection

**Table:**

Oxford Diffraction Xcalibur diffractometer with a Sapphire CCD detector	2174 independent reflections
Radiation source: fine-focus sealed tube	1326 reflections with *I* > 2σ(*I*)
graphite	*R*_int_ = 0.027
Rotation method data acquisition using ω and φ scans	θ_max_ = 25.4°, θ_min_ = 2.6°
Absorption correction: multi-scan (*CrysAlis RED*; Oxford Diffraction, 2009)	*h* = −8→9
*T*_min_ = 0.944, *T*_max_ = 0.984	*k* = −18→19
4285 measured reflections	*l* = −12→5

### Refinement

**Table:**

Refinement on *F*^2^	Primary atom site location: structure-invariant direct methods
Least-squares matrix: full	Secondary atom site location: difference Fourier map
*R*[*F*^2^ > 2σ(*F*^2^)] = 0.058	Hydrogen site location: inferred from neighbouring sites
*wR*(*F*^2^) = 0.139	H atoms treated by a mixture of independent and constrained refinement
*S* = 1.04	*w* = 1/[σ^2^(*F*_o_^2^) + (0.0552*P*)^2^ + 0.6042*P*] where *P* = (*F*_o_^2^ + 2*F*_c_^2^)/3
2174 reflections	(Δ/σ)_max_ < 0.001
149 parameters	Δρ_max_ = 0.24 e Å^−^^3^
1 restraint	Δρ_min_ = −0.18 e Å^−^^3^

### Special details

**Table:**

Geometry. All e.s.d.'s (except the e.s.d. in the dihedral angle between two l.s. planes) are estimated using the full covariance matrix. The cell e.s.d.'s are taken into account individually in the estimation of e.s.d.'s in distances, angles and torsion angles; correlations between e.s.d.'s in cell parameters are only used when they are defined by crystal symmetry. An approximate (isotropic) treatment of cell e.s.d.'s is used for estimating e.s.d.'s involving l.s. planes.
Refinement. Refinement of *F*^2^ against ALL reflections. The weighted *R*-factor *wR* and goodness of fit *S* are based on *F*^2^, conventional *R*-factors *R* are based on *F*, with *F* set to zero for negative *F*^2^. The threshold expression of *F*^2^ > σ(*F*^2^) is used only for calculating *R*-factors(gt) *etc*. and is not relevant to the choice of reflections for refinement. *R*-factors based on *F*^2^ are statistically about twice as large as those based on *F*, and *R*- factors based on ALL data will be even larger.

### Fractional atomic coordinates and isotropic or equivalent isotropic displacement parameters (Å^2^)

**Table:**

	*x*	*y*	*z*	*U*_iso_*/*U*_eq_	
C1	0.5932 (5)	0.5531 (2)	0.2952 (4)	0.0497 (9)	
C2	0.6538 (6)	0.5479 (3)	0.4421 (4)	0.0737 (13)	
H2	0.6270	0.5905	0.4942	0.088*	
C3	0.7543 (6)	0.4790 (3)	0.5116 (4)	0.0764 (13)	
H3	0.7974	0.4766	0.6109	0.092*	
C4	0.7924 (5)	0.4137 (3)	0.4374 (4)	0.0618 (10)	
C5	0.7280 (6)	0.4193 (3)	0.2923 (5)	0.0729 (12)	
H5	0.7494	0.3752	0.2401	0.088*	
C6	0.6318 (5)	0.4888 (3)	0.2201 (4)	0.0659 (11)	
H6	0.5933	0.4920	0.1211	0.079*	
C7	0.6778 (4)	0.7666 (2)	0.3447 (3)	0.0426 (8)	
C8	0.7768 (5)	0.8427 (2)	0.3252 (4)	0.0514 (9)	
H8A	0.6928	0.8786	0.2525	0.062*	
H8B	0.8681	0.8245	0.2902	0.062*	
C9	0.8661 (5)	0.8942 (2)	0.4590 (3)	0.0502 (9)	
H9A	0.9560	0.8596	0.5299	0.060*	
H9B	0.7765	0.9099	0.4974	0.060*	
C10	0.9552 (5)	0.9732 (2)	0.4338 (3)	0.0472 (9)	
H10A	0.8651	1.0072	0.3619	0.057*	
H10B	1.0450	0.9572	0.3958	0.057*	
C11	0.8969 (6)	0.3379 (3)	0.5150 (5)	0.0877 (14)	
H11A	0.8168	0.2989	0.5336	0.132*	
H11B	0.9882	0.3555	0.6040	0.132*	
H11C	0.9525	0.3109	0.4572	0.132*	
N1	0.6017 (4)	0.71857 (18)	0.2220 (3)	0.0503 (8)	
H1N	0.622 (5)	0.730 (2)	0.147 (3)	0.060*	
O1	0.3945 (4)	0.62103 (16)	0.0532 (2)	0.0675 (8)	
O2	0.3391 (3)	0.66139 (16)	0.2692 (3)	0.0676 (8)	
O3	0.6643 (3)	0.74728 (14)	0.4557 (2)	0.0530 (7)	
S1	0.46037 (13)	0.63862 (6)	0.20264 (10)	0.0530 (3)	

### Atomic displacement parameters (Å^2^)

**Table:**

	*U*^11^	*U*^22^	*U*^33^	*U*^12^	*U*^13^	*U*^23^
C1	0.058 (2)	0.046 (2)	0.046 (2)	−0.0189 (18)	0.0211 (19)	−0.0049 (17)
C2	0.121 (4)	0.052 (3)	0.048 (2)	−0.004 (3)	0.032 (2)	−0.005 (2)
C3	0.115 (4)	0.061 (3)	0.044 (2)	−0.007 (3)	0.020 (2)	0.001 (2)
C4	0.060 (2)	0.064 (3)	0.064 (3)	−0.009 (2)	0.026 (2)	0.000 (2)
C5	0.083 (3)	0.074 (3)	0.067 (3)	0.013 (3)	0.034 (2)	−0.009 (2)
C6	0.077 (3)	0.073 (3)	0.046 (2)	0.005 (2)	0.022 (2)	−0.006 (2)
C7	0.050 (2)	0.038 (2)	0.040 (2)	−0.0043 (17)	0.0181 (17)	−0.0070 (15)
C8	0.065 (2)	0.043 (2)	0.047 (2)	−0.0127 (18)	0.0215 (18)	−0.0077 (16)
C9	0.057 (2)	0.039 (2)	0.049 (2)	−0.0055 (18)	0.0142 (17)	−0.0054 (16)
C10	0.049 (2)	0.038 (2)	0.047 (2)	−0.0009 (17)	0.0093 (17)	−0.0032 (15)
C11	0.084 (3)	0.081 (4)	0.096 (4)	0.018 (3)	0.033 (3)	0.018 (3)
N1	0.072 (2)	0.0452 (17)	0.0398 (16)	−0.0234 (16)	0.0283 (16)	−0.0108 (14)
O1	0.0841 (19)	0.0595 (18)	0.0454 (14)	−0.0210 (14)	0.0091 (13)	−0.0094 (12)
O2	0.0662 (17)	0.0630 (18)	0.0816 (19)	−0.0098 (14)	0.0372 (16)	0.0012 (14)
O3	0.0784 (18)	0.0485 (15)	0.0385 (13)	−0.0094 (13)	0.0292 (13)	−0.0069 (11)
S1	0.0622 (6)	0.0473 (6)	0.0471 (5)	−0.0178 (5)	0.0179 (4)	−0.0062 (4)

### Geometric parameters (Å, °)

**Table:**

C1—C6	1.374 (5)	C8—H8A	0.9700
C1—C2	1.378 (5)	C8—H8B	0.9700
C1—S1	1.757 (4)	C9—C10	1.510 (4)
C2—C3	1.379 (6)	C9—H9A	0.9700
C2—H2	0.9300	C9—H9B	0.9700
C3—C4	1.379 (5)	C10—C10^i^	1.515 (6)
C3—H3	0.9300	C10—H10A	0.9700
C4—C5	1.360 (5)	C10—H10B	0.9700
C4—C11	1.503 (6)	C11—H11A	0.9600
C5—C6	1.383 (6)	C11—H11B	0.9600
C5—H5	0.9300	C11—H11C	0.9600
C6—H6	0.9300	N1—S1	1.661 (3)
C7—O3	1.206 (3)	N1—H1N	0.851 (18)
C7—N1	1.384 (4)	O1—S1	1.425 (2)
C7—C8	1.498 (4)	O2—S1	1.423 (3)
C8—C9	1.507 (4)		
			
C6—C1—C2	119.3 (4)	C8—C9—C10	113.0 (3)
C6—C1—S1	119.7 (3)	C8—C9—H9A	109.0
C2—C1—S1	121.0 (3)	C10—C9—H9A	109.0
C3—C2—C1	119.7 (4)	C8—C9—H9B	109.0
C3—C2—H2	120.2	C10—C9—H9B	109.0
C1—C2—H2	120.2	H9A—C9—H9B	107.8
C2—C3—C4	121.6 (4)	C9—C10—C10^i^	114.3 (3)
C2—C3—H3	119.2	C9—C10—H10A	108.7
C4—C3—H3	119.2	C10^i^—C10—H10A	108.7
C5—C4—C3	117.7 (4)	C9—C10—H10B	108.7
C5—C4—C11	121.6 (4)	C10^i^—C10—H10B	108.7
C3—C4—C11	120.7 (4)	H10A—C10—H10B	107.6
C4—C5—C6	121.9 (4)	C4—C11—H11A	109.5
C4—C5—H5	119.0	C4—C11—H11B	109.5
C6—C5—H5	119.0	H11A—C11—H11B	109.5
C1—C6—C5	119.7 (4)	C4—C11—H11C	109.5
C1—C6—H6	120.1	H11A—C11—H11C	109.5
C5—C6—H6	120.1	H11B—C11—H11C	109.5
O3—C7—N1	122.0 (3)	C7—N1—S1	125.0 (2)
O3—C7—C8	124.5 (3)	C7—N1—H1N	121 (2)
N1—C7—C8	113.6 (3)	S1—N1—H1N	114 (2)
C7—C8—C9	114.3 (3)	O2—S1—O1	120.31 (18)
C7—C8—H8A	108.7	O2—S1—N1	108.08 (15)
C9—C8—H8A	108.7	O1—S1—N1	103.64 (15)
C7—C8—H8B	108.7	O2—S1—C1	109.20 (16)
C9—C8—H8B	108.7	O1—S1—C1	108.66 (17)
H8A—C8—H8B	107.6	N1—S1—C1	105.98 (16)
			
C6—C1—C2—C3	1.0 (6)	C8—C9—C10—C10^i^	179.5 (4)
S1—C1—C2—C3	178.5 (3)	O3—C7—N1—S1	9.4 (5)
C1—C2—C3—C4	−1.7 (7)	C8—C7—N1—S1	−170.9 (3)
C2—C3—C4—C5	0.3 (6)	C7—N1—S1—O2	40.5 (3)
C2—C3—C4—C11	−177.9 (4)	C7—N1—S1—O1	169.2 (3)
C3—C4—C5—C6	1.8 (6)	C7—N1—S1—C1	−76.5 (3)
C11—C4—C5—C6	180.0 (4)	C6—C1—S1—O2	139.5 (3)
C2—C1—C6—C5	1.0 (6)	C2—C1—S1—O2	−38.0 (4)
S1—C1—C6—C5	−176.5 (3)	C6—C1—S1—O1	6.5 (3)
C4—C5—C6—C1	−2.4 (6)	C2—C1—S1—O1	−170.9 (3)
O3—C7—C8—C9	1.7 (5)	C6—C1—S1—N1	−104.3 (3)
N1—C7—C8—C9	−178.0 (3)	C2—C1—S1—N1	78.2 (3)
C7—C8—C9—C10	−176.5 (3)		

Symmetry codes: (i) −*x*+2, −*y*+2, −*z*+1.

### Hydrogen-bond geometry (Å, °)

**Table:**

*D*—H···*A*	*D*—H	H···*A*	*D*···*A*	*D*—H···*A*
N1—H1N···O3^ii^	0.85 (2)	2.12 (2)	2.968 (3)	177 (3)

Symmetry codes: (ii) *x*, −*y*+3/2, *z*−1/2.
